# Supplementary figures and images for: The measurement of self-regulation in the Adolescent Brain Cognitive Development (ABCD) Study
Source: PLoS One. 2025 May 5;20(5):e0322795. doi: 10.1371/journal.pone.0322795 (PMC12052097; doi:10.1371/journal.pone.0322795)

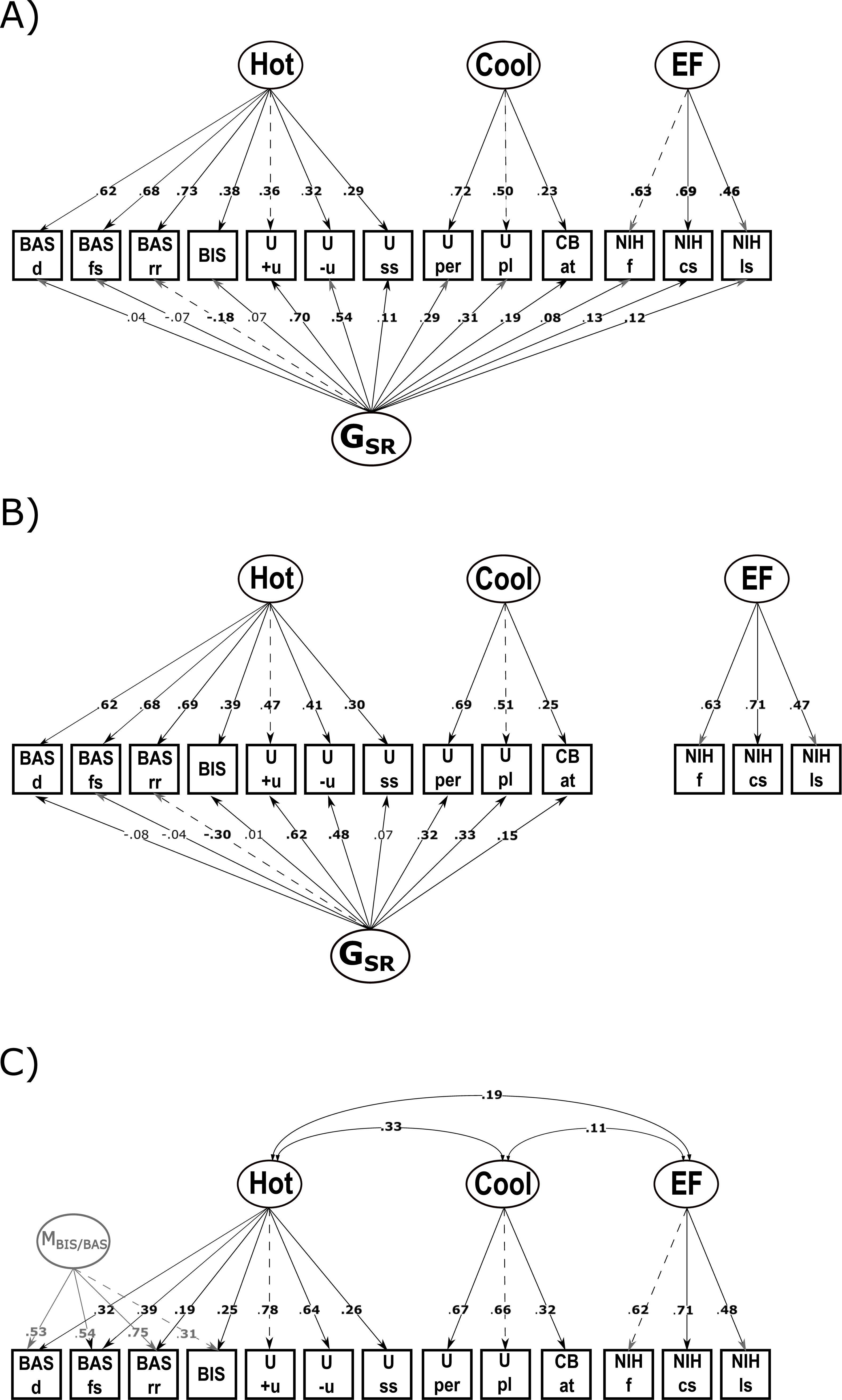

Supplement: S1 Fig — Schematic representation of all three defensible measurement models of SR with standardized factor loadings. Reference indicators are depicted with dashed lines. (A) Model 1 (B) Models 2a and 2b (C) Model 3b; Hot/Cool/EF: domain-specific factors for hot/cool/executive functions components of SR; MBIS/BAS: method factor for BIS/BAS indicators; U: UPPS-P; + u: positive urgency; -u: negative urgency; ss: sensation seeking; pl: planning; per: perseverance; BIS/BAS: Behavioral Inhibition and Behavioral Approach System Scale, inhibition; rr: reward responsivity; fs: fun seeking; d: drive; CB: Parent Child Behavior Check List; at: attention problems; NIH: NIH toolbox behavioral tasks; f: flanker task; cs: dimensional card sort task; ls: list sort working memory test. (TIF) [file pone.0322795.s001.tif]

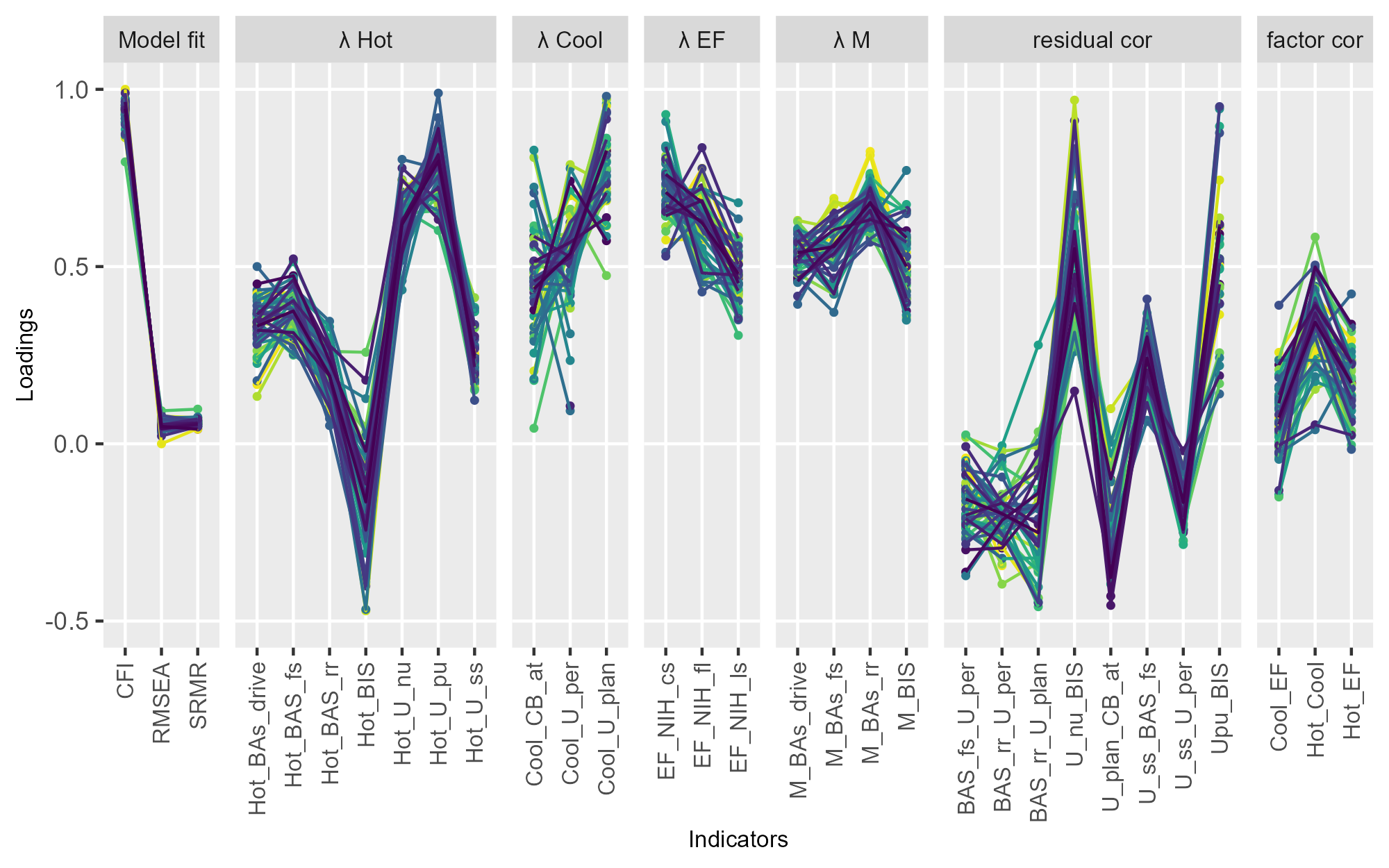

Supplement: S2 Fig — Factor loadings of the full dataset per factor (full) are compared to factor loadings of cross-validation sets (CV 1-CV 50) to assess the robustness of the factor structure over smaller samples. Fit: model fit; load_Hot/Cool/EF: factor loadings onto domain specific factors for hot/cool/executive function components of self-regulation (SR); load_M: factor loadings onto method factor for BIS/BAS scales; res_cor: residual correlations; factor_cor: correlations between factors; U: UPPS-P; + u: positive urgency; -u: negative urgency; ss: sensation seeking; pl: planning; per: perseverance; BIS/BAS: Behavioral Inhibition and Behavioral Approach System Scale; rr: reward responsivity; fs: fun seeking; d: drive; CB: Parent Child Behavior Check List; rb: rule breaking behavior; ag: aggressive behavior; th: thought problems; at: attention problems; NIH: NIH toolbox behavioral tasks; f: flanker task; cs: dimensional card sort task; ls: list sort working memory test. (TIF) [file pone.0322795.s002.tif]
