# Supplementary material for: The measurement of self-regulation in the Adolescent Brain Cognitive Development (ABCD) Study
Source: PLoS One. 2025 May 5;20(5):e0322795. doi: 10.1371/journal.pone.0322795 (PMC12052097; doi:10.1371/journal.pone.0322795)
Supplement: Attachment — Submitted filename: Response to Reviewers.docx [file pone.0322795.s004.docx]

Dear Prof. Dr. Iftikhar Ahmed Khan, dear Reviewer Dr. Grass,

Thank you for handling and reviewing our manuscript, number PONE-D-24-12171, and for your insightful and positive feedback! We appreciate the constructive comments on how to improve the manuscript. We have carefully considered all comments and incorporated them into the revised manuscript. A version of the revised manuscript without track changes is also provided. Please find below a point-by-point response to the reviewer’s comments. Line numbers refer to the final manuscript without track changes.

Yours sincerely,

Merle Marek, Axel Heep and Andrea Hildebrandt

**Journal Requirements:**

1. When submitting your revision, we need you to address these additional requirements.

Please ensure that your manuscript meets PLOS ONE's style requirements, including those for file naming. The PLOS ONE style templates can be found at <https://journals.plos.org/plosone/s/file?id=wjVg/PLOSOne_formatting_sample_main_body.pdf> and <https://journals.plos.org/plosone/s/file?id=ba62/PLOSOne_formatting_sample_title_authors_affiliations.pdf2>. We note that the grant information you provided in the ‘Funding Information’ and ‘Financial Disclosure’ sections do not match.

We sincerely thank the editor for the valuable and constructive comments. In response, we have adjusted the size of the figures and updated the panel labels in the figure legends as suggested. Additionally, we have modified the settings with PACE, reformatted the tables, and aligned the title page with the journal’s requirements. We appreciate the opportunity to improve the manuscript and are confident these adjustments enhance its clarity and presentation.

When you resubmit, please ensure that you provide the correct grant numbers for the awards you received for your study in the ‘Funding Information’ section.

Thank you for pointing this out. We understand the confusion regarding the grant information. To clarify, the authors of this study did not receive any direct grant funding; however, the ABCD study, from which data were utilized, did. Upon resubmission, we will adapt the funding section to include the relevant grant numbers for the ABCD study, ensuring accurate attribution.

3. When completing the data availability statement of the submission form, you indicated that you will make your data available on acceptance. We strongly recommend all authors decide on a data sharing plan before acceptance, as the process can be lengthy and hold up publication timelines. Please note that, though access restrictions are acceptable now, your entire data will need to be made freely accessible if your manuscript is accepted for publication. This policy applies to all data except where public deposition would breach compliance with the protocol approved by your research ethics board. If you are unable to adhere to our open data policy, please kindly revise your statement to explain your reasoning and we will seek the editor's input on an exemption. Please be assured that, once you have provided your new statement, the assessment of your exemption will not hold up the peer review process.

We have revised the statement and added a statement in line 330-335 of our manuscript:

‘Due to data access restrictions outlined by the ABCD study, the authors are unable publicly share the study’s dataset. However, the ABCD study embraces an open science approach and data access can be requested by any eligible researcher with a valid research use of the data. Please note that the full analysis code can be accessed at <https://osf.io/nzjf7/?view_only=ebd4dfaa5c8b4f0fb499b6071f43831d> .’

We appreciate your guidance on this matter and are confident this revised statement adheres to the journal’s policy while providing clear instructions for researchers seeking data access.

4. We notice that your supplementary figures are uploaded with the file type 'Figure'. Please amend the file type to 'Supporting Information'. Please ensure that each Supporting Information file has a legend listed in the manuscript after the references list.

Thank you for bringing this to our attention. We have addressed this by uploading the figures as 'Supporting Information,' ensuring that each supplementary figure is accompanied by a detailed legend within the manuscript.

Reviewers' comments:

Reviewer's Responses to Questions

**Comments to the Author**

1. Is the manuscript technically sound, and do the data support the conclusions?

The manuscript must describe a technically sound piece of scientific research with data that supports the conclusions. Experiments must have been conducted rigorously, with appropriate controls, replication, and sample sizes. The conclusions must be drawn appropriately based on the data presented.

Reviewer #1: Yes

2. Has the statistical analysis been performed appropriately and rigorously?

Reviewer #1: Yes

3. Have the authors made all data underlying the findings in their manuscript fully available?

The [PLOS Data policy](http://track.editorialmanager.com/CL0/http:%2F%2Fwww.plosone.org%2Fstatic%2Fpolicies.action%23sharing/1/010f0193b2fa538b-36a72850-d182-4b97-9558-d0d8e6c87070-000000/sS3iMJmkH7ahj_1gUtmlg27x6jW0b7qKByABykB4VZs=189) requires authors to make all data underlying the findings described in their manuscript fully available without restriction, with rare exception (please refer to the Data Availability Statement in the manuscript PDF file). The data should be provided as part of the manuscript or its supporting information, or deposited to a public repository. For example, in addition to summary statistics, the data points behind means, medians and variance measures should be available. If there are restrictions on publicly sharing data—e.g. participant privacy or use of data from a third party—those must be specified.

Reviewer #1: No

Please refer to our previous response to the editor’s third comment regarding data sharing issues.

4. Is the manuscript presented in an intelligible fashion and written in standard English?

PLOS ONE does not copyedit accepted manuscripts, so the language in submitted articles must be clear, correct, and unambiguous. Any typographical or grammatical errors should be corrected at revision, so please note any specific errors here.

Reviewer #1: Yes

5. Review Comments to the Author

Please use the space provided to explain your answers to the questions above. You may also include additional comments for the author, including concerns about dual publication, research ethics, or publication ethics. (Please upload your review as an attachment if it exceeds 20,000 characters)

**Reviewer #1:** The submitted manuscript reports an interdisciplinary approach to research related to self-regulation dealing with significant assessment issues. The aim of developing a measurement model for assessing self-regulation within the ABCD study could have large impact because of the widespread use of ABCD data in brain research. Because I am no expert in the field of neuroscience, my recommendations mainly refer to a differential/assessment perspective and to issues of research on self-regulation in general.

However, some aspects of the manuscript could be improved as explained in the following:
- Structure: from a theoretical perspective, the paper is about the valid assessment of self-regulation in a specific data repository considering instruments implemented there. Because the basis of assessment should be the theoretical conceptualization of a construct, I suggest to focus on the conceptualization of SR first, before introducing the ABCD study more in detail as context for the current research project. In some parts of the introduction, the structure could be improved with subheadings referring to self-regulation and the ABCD study as well as with self-regulation tasks within the ABCD study ("Present study"). Currently, those contents are partly mixed up what can lead to reduced comprehension by readers.

We sincerely thank the reviewer for the valuable feedback, particularly regarding the structure and theoretical framing of the introduction. In response to the suggestions, we have revised the structure to improve the flow and added subheadings to enhance clarity. These subheadings now highlight the conceptualization of self-regulation, general measurement issues, and the specific context of self-regulation in the ABCD dataset.

To address the reviewer's concern, we reorganized the paragraph ‘The measurement of self-regulation’ (lines 111–132) so that the ABCD study is discussed only at the end of the paragraph (lines 127–132). This change serves as a bridge to the following section, ‘The measurement of self-regulation in the ABCD study’ clarifying that the initial discussion focuses on measurement challenges in general. Additionally, we now introduce the measures of self-regulation in the ABCD study earlier in the manuscript (lines 147–153, within the paragraph ‘The measurement of self-regulation in the ABCD study’). This is to clarify the selection of the defensible models and to enhance the narrative's coherence. The new text reads:

‘We evaluated all available instruments of the ABCD study related to mental health and neurocognition based on their theoretical overlap with the construct of SR or its subconstructs. Potentially related constructs were identified based on [2] and [30], which include a glossary of major terms and mental processes related to SR and an overview of task and survey-based indicators of SR, respectively. We identified three questionnaires measuring hot and cool facets of self-reported impulsivity and inhibition/reward seeking, as well as caregiver-reported attention problems, and three EF tasks as potential indicators of SR.’

We carefully reflected on the suggestion to prioritize the conceptualization of self-regulation before introducing the dataset. This approach is undoubtedly essential to most psychometric studies, where the theoretical framework typically informs the selection of measures. However, our study takes a different perspective: rather than beginning with a theoretical conceptualization and subsequently choosing corresponding indicators to be applied, we were restricted to start with the predefined measures available in the ABCD dataset and our goal was to explore whether they can meaningfully align with the dual process model of self-regulation. We are concerned that adopting a structure where the theoretical framework precedes the dataset could mislead readers to assume that we first conceptualized self-regulation and then derived measures to be used to test the taxonomic/psychometric model. To avoid this potential misunderstanding, we believe it is critical to emphasize from the outset that our focus is on evaluating the suitability of existing measures within the dataset, rather than operationalizing self-regulation beginning from theory. While the suggested structure offers clear advantages in contexts where theoretically derived measurement models allow flexibility in indicator selection, we believe our approach better avoids potential misinterpretation and more accurately reflects the unique goals of our research. We hope these revisions and our rationale address the reviewer's points while maintaining the study's focus.

- in the introduction, the motivation for/implications of studying brain-behavior associations should become clear because this is one important context and starting point of the reported research.

Thank you for pointing out the importance of highlighting the motivation and implications of studying brain-behavior associations in the introduction. To address this, we have added the sentence in lines 49–51:

‘Understanding the link between brain systems and networks and human behavior is central to cognitive-behavioral neuroscience and offers critical insights into mechanisms underlying cognition, personality, and psychiatric conditions.’

- l.43: a priori

We have corrected the typo in line 53.

- l. 47/48: the sentence confuses two aspects: validity of used instruments and measurement models for representing latent constructs. Both are important issues when brain-behavior associations (and psychological variables in general) are examined

Thank you for this careful observation. We agree that our previous phrasing might have suggested that modeling is directly related to validity of tests. Instead of referring to ‘enhancing psychometrically sound research’, we now refer to ‘improve the statistical analysis of brain-behavior associations’ in line 55-59 and improved the sentence structure to enhance the reading flow:

‘As the expertise of researchers analyzing data from these neuroimaging initiatives is often in neuroimaging but less so in psychometrics, proposals for psychometric models assessed for robustness, together with code for implementation, have the potential to improve the statistical analysis of brain-behavior associations.’

- l. 48: I suggest to use "research on" instead of "tests"

We agree with the suggestion, however, this part was deleted from the text due to the previous suggestion.

- l. 50 - l. 54: These sentences use present tense. For consistency when referring to the reported research, past tense should be used. Additionally, to me the intention of this part was not completely clear. If the authors wanted to briefly outline the aims of the reported research, I recommend to clearly state that they are writing about the aims. Taking into account my suggestion referring to the structure of the introduction this would be placed after referring to self-regulation in the introduction.

We have revised this section to use past tense for consistency when referring to the reported research. Additionally, we have explicitly clarified that this part outlines the goals of our study. The updated text (lines 92–63) now reads:

‘The aim of this study was to foster a well-grounded and consistent construct representation of SR in future brain-behavior association studies in the ABCD dataset – today's largest open longitudinal neuroimaging study. To reach this goal, we investigated alternative measurement models and provided code that can be used in future analyses of SR’s neural correlates.’

Regarding the placement of this part, we respectfully refer to our first comment, where we explain our rationale for the structure of the introduction.

- l. 55: for a sound introduction, for me, an overview about definitions and the nomological network of self-regulation was missing when it was introduced. what are commonalities of definitions and what are shortcomings the authors write about? This could include aspects following later, e.g., in ll. 84

We have expanded the introduction with a more elaborate definition of the construct of self-regulation in lines 66–84. This revised section now addresses the commonalities among definitions (lines 66–79), as well as key shortcomings in the field, such as the jingle-jangle fallacy, which often arises in self-regulation research (lines 79–81). We also discuss the diversity of the postulated self-regulation subconstructs (lines 69–73):

‘SR refers to the ability to actively guide oneself toward a desired emotional, behavioral, or cognitive state (e.g., [1]). It involves adapting internal states (i.e. emotions and cognitions) and behaviors to align with contextual demands [2]. Context, in this regard, encompasses situational factors, social expectations, and personal goals. SR is a complex, multidimensional construct that depends on dynamic interactions between traits (e.g., temperament, impulsivity) and states (e.g., hormonal and physiological conditions) and involves various subskills, such as delay of gratification, executive functions (EFs) and self-control (see [2] for a glossary of related terminology). Although SR and self-control are sometimes used interchangeably, they are more accurately understood as distinct but related concepts. Self-control specifically addresses behavioral regulation in the face of conflicts between competing goals, such as resisting short-term rewards to prioritize long-term outcomes. In contrast, SR encompasses a broader set of processes, including planning, goal-setting, monitoring, in addition to the execution of behavior [1,3,4]. Unlike self-control, SR is not necessarily dependent on the presence of a conflict between competing goals [1]. Given this complexity, SR has been challenging to define consistently, often falling prey to jingle-jangle fallacies, where similar terms are used interchangeably or distinct concepts are conflated (e.g., [5]). Despite definitional challenges, SR has drawn significant interest across biological, differential, developmental, and clinical psychology due to its malleability [6] and its predictive power for life outcomes such as academic achievement, social relationship quality, and substance-use behavior [7,8].’

To avoid redundancy, we removed the later discussion of self-regulation subconstructs from the Method section. These changes aim to provide a clearer and more cohesive introduction to self-regulation, as recommended.

- l. 57: "biological-" and so on should be written without hyphen

We have corrected the typo in line 82.

- l. 61: for readers it would be helpful to define what "context" means and to refer also to the role individual goals have related to self-regulation

We clarified the term in lines 68-69:

‘Context, in this regard, encompasses situational factors, social expectations, and personal goals.’

And added the role of personal goals in lines 74-77:

‘Self-control specifically addresses behavioral regulation in the face of conflicts between competing goals, such as resisting short-term rewards to prioritize long-term outcomes. In contrast, SR encompasses a broader set of processes, including planning, goal-setting, monitoring, in addition to the execution of behavior [1,3,4].’

- l. 62: for understanding it would be beneficial to explain the relation between self-control (probably referred to by the authors when writing about behavioral conflict) and self-regulation

We added an explanation of the distinction between the two concepts in lines 73-79:

‘Although SR and self-control are sometimes used interchangeably, they are more accurately understood as distinct but related concepts. Self-control specifically addresses behavioral regulation in the face of conflicts between competing goals, such as resisting short-term rewards to prioritize long-term outcomes. In contrast, SR encompasses a broader set of processes, including planning, goal-setting, monitoring, in addition to the execution of behavior [1,3,4]. Unlike self-control, SR is not necessarily dependent on the presence of a conflict between competing goals [1].’

- l. 65: when referring to the dual-process theory the authors name domain-specific facets which could be understood in a misleading way. maybe it would be clearer to refer to classes of processes or to two systems complementing each other

We deleted the word ‘facets’ throughout the manuscript and replaced it with ‘systems’ (e.g., lines 87, 156, Table 1, 500), when discussing the dual process theory. When measurement models are being discussed, we substituted it with ‘factors’ (e.g., lines 510, 527).

- l. 67: because EF are central to this research, the three EF introduced should be explained briefly

We extended the paragraph and elaborated on the three EFs lines 89-95:

‘EFs form an integral part of this cool components, as they enable the cognitive control necessary for effective SR. However, EFs are not synonymous with SR; while critical, they are not sufficient for SR and can also support other psychological goals [2,12]. The factor structure of EF, detailed in [11], comprises three core components: cognitive flexibility or set shifting, which involves the ability to switch between tasks, mental sets or operations; working memory, which allows active updating, operating, and monitoring of mental representations; and inhibitory control, the deliberate suppression of prepotent responses.’

- when referring to measurement approaches and the structure of self-regulation, previous research on convergent and discriminant validity should be considered, because differences between tasks refer not only to kind of measure but also to the subconstruct measured, see e.g., Duckworth & Kern (2011) <https://doi.org/10.1016/j.jrp.2011.02.004>

We thank the reviewer for her valuable feedback and added the reference ([23]) in lines 112-116:

‘Efforts to establish a comprehensive nomological network model for SR have provided valuable insights (e.g., [21,22]), but the complexity of this task remains significant. Meta-analytic research on the convergent validity of SR measures reveals weak correlations between different content domains, such as between EF tasks or between EF tasks and delay of gratification measures [23].’

This addition emphasizes the broader challenges of achieving convergent validity in self-regulation research. For further changes related to convergent validity, please also refer to our response to the next comment.

- l. 91: "content validity" - I guess, the authors mean construct validity?

Thank you for pointing out the potential for confusion in our use of the term ‘content validity’. We acknowledge that the previous phrasing may have implied construct validity, whereas we were specifically referring to content validity. To clarify, we revised the sentence in lines 119–121 to explicitly reference the heterogeneity of content within the construct. This revision not only clarifies our focus on content validity but also serves to connect content and construct (convergent) validity, which were addressed earlier in the text, to different assessment domains. The updated sentence now reads:

‘Heterogeneous indicators of SR from various content domains – which are often assessed using different measurement approaches – enhance content validity, but correlations between survey and behavioral data of SR are notably small [21].’

- l. 97: the authors should briefly explain why behavioral (experimental?) tasks are advantageous despite low reliability, which theoretically implies low validity, too. They should explain why they are better suited for studying brain-behavior relationships.

We added a paragraph to clarify this point in lines 125-128:

‘They also typically assess more isolated constructs (e.g. response inhibition), which often correspond to lower variability in brain correlates. Therefore, behavioral tasks may be more appropriate for studying brain-behavior relationships [29], which is one of the main objectives of the ABCD dataset.’

Present Study: The authors could argue more transparently why they decided to test the three models and what tasks were considered to load on them. From a theoretical point of view, I have not completely understood, why EF were considered to mirror a factor distinct from (hot)/cool system on the same hierarchical level. Therefore, naming example tasks would be helpful when introducing EF and hot/cool processes above.

Thank you for highlighting the need for greater transparency in explaining our decision to test the three models and the tasks considered in this process. In response, we added an overview of the models to provide a clearer rationale for their selection and progression. This overview appears in lines 168–176 and reads:

‘Based on the theoretical assumption that all selected measures indicate – at least to some extent - the general construct of SR, our initial model included a general factor that reflected potential common variance of all indicators in addition to the factors representing domain-specific traits. However, recognizing the challenges in achieving convergent validity across different measurement modalities – specifically self- and other-reports as compared with tasks – we progressively relaxed this assumption. In Model 2, we separated behavioral tasks from survey-based measures while retaining a general factor. Finally, we tested a model without a general SR factor, allowing for the possibility that SR might be better represented without a general construct. The three models that were tested are shown in Fig 1 and described in more detail here:’

Additionally, we expanded the description of indicators’ loading on the factors in lines 241–266. For further details on the categorization of indicators according to the dual-process theory, please refer to our response to the comment addressing the description of the tasks.

To further clarify why EFs are represented as a distinct factor, we added a paragraph in the section ‘The measurement of self-regulation in the ABCD study’ (lines 147–153, please refer to our comment regarding the structure of the Introduction for further detail). This paragraph explains that EFs are assessed using behavioral tasks, while hot and cool processes are derived from survey data, justifying their separate representation in the models. We hope these revisions address the reviewer's concerns and enhance the clarity of our theoretical framework and methodological choices.

--> l. 137 referring to different assessment domains is not clear to readers who do not know the ABCD tasks because EF and cool processes are often measured as behavioral tasks.

Thank you for pointing out the potential confusion regarding the assessment domains in the ABCD study. To clarify this distinction, we introduced a paragraph earlier in the manuscript (lines 147–153) that provides an overview of the SR measures used in the ABCD dataset. For further details, please refer to our responses addressing the structure of the Introduction and the transparency of indicator selection.

Additionally, we rephrased the relevant section to make it more clear that we are specifically referring to the indicator set in the ABCD study when discussing the relationship between Cool factors and surveys, and EF factors and task data. The revised text (lines 181–184) now reads:

‘Even though the Cool and EF factors are conceptually closely related according to the dual-process theory, due to differing assessment domains in the ABCD study (tasks for EFs and surveys for other cool indicators), we expected their correlation to be too small to be combined within a common factor [21].’

For introducing tested models 1 to 3 a figure displaying them would improve the clarity of this part.

We added a figure (Fig 1) with a schematic representation of all three defensible models.

Methods:
- l. 180: what is the reference [4] referring to? On what basis did the authors choose tasks potentially measuring self-regulation. For transparency and reproducibility of results more details are needed.

To address this suggestion, we elaborated on our procedure in an additional paragraph in the Introduction (lines 147–153), as explained in our earlier comment on the structure of the Introduction. This paragraph clarifies which measures are included in the ABCD study and how they are assessed, now providing readers with this information earlier in the manuscript.

Additionally, we included a reference [30] in the Instruments paragraph of the Method section (line 239) to further detail the rationale of our selection process. We hope these revisions enhance the transparency and reproducibility of our approach.

- Similarly, more explanation is needed, on what basis have the authors decided what instruments are used as indicators for hot/cool system/EF?

We deleted a previous sentence and elaborated on this decision in lines 268-275:

‘Individual scale scores from these instruments were categorized based on the dual-process theory as outlined in [1]. Measures capturing rapid, bottom-up responses to high incentive stimuli – such as sensation seeking, fun seeking, reward responsiveness and drive – alongside emotion-laden impulsivity (positive and negative urgency, inhibition) were classified as components of the hot SR system. In contrast, indicators of deliberate, top-down processes including planning, perseverance, and attention problems were attributed to the cool SR system. Given their reliance on cognitive control mechanisms, the three EF indicators were categorized as part of the cool component.’

- l. 184: UPPS-P is an abbreviation that has not been introduced to readers.

Thank you for bringing this to our attention. We have clarified the origin of the UPPS-P abbreviation in lines 241–243 by adding the following explanation:

‘First, the UPPS-P ((negative) urgency, premeditation, perseverance, sensation seeking – positive urgency) questionnaire [33,34] was selected, which captures impulsivity on five scales with four items each: […]’

- in general, the usage of many abbreviations for considered tasks are not easy to follow. If future readers should mainly consist of researchers familiar with ABCD and those abbreviations are common in this context, they could remain.

Thank you for this valuable feedback regarding the use of abbreviations. To improve readability, we removed abbreviations from the text where possible, such as when introducing the indicators in lines 238–267 (‘Measurement instruments’). In cases where abbreviations were previously used, we substituted them with the full scale names, such as in the ‘Statistical Analysis’ section (lines 297–310). Additionally, we replaced abbreviations with full scale names in Table 1 and Table 2. In the Results section (lines 338–458), we summarized factor loadings and removed the abbreviations of the individual scales to enhance clarity. For the figures, we retained abbreviations to ensure their readability but provided full explanations of these abbreviations in the figure notes. We hope these revisions maintain readability and lead to enhanced accessibility for readers.

- Tasks considered in this research should be described briefly in text or in table 1 with example tasks/items, number of tasks/items etc.

To enhance understanding, we added example items and the number of items per scale in the text (lines 241–266). Additionally, we moved the reference to Table 1, which lists all indicators of self-regulation and outcome variables, to immediately follow the description of the indicators (lines 266–267). This adjustment helps consolidate information about the indicators and directs readers to Table 1 for further details if needed.

- Do abbreviations in Table 1 refer to variable names used in ABCD data? otherwise, they are rather confusing. Abbreviations such as "CB_at" should be left out in table 1 or explained in a table note because acc. to APA table content should be understood on its own so that abbreviations have to be explained again in each table.

Please refer to the comment above (‘in general, the usage of many abbreviations […]’).

- ll. 231: What were criteria for splitting the sample into subsamples, were them randomly assigned to subsamples besides considering family membership?

In line 318, we added

‘Participants were then randomly allocated to the CV sets ensuring…’.

- in general, introducing subheadings for the description of sample, procedure, instruments, and statistical analysis would improve the structure of the method section.

For a better structure of the section, we added subheadings in lines 216 (Participants), 237 (Measurement instruments), and 296 (Statistical analysis).

Results:
- l. 254: "is mostly determined" should be past tense

We corrected the tense in line 341.

- Capturing the results at first glance would be supported by providing figures for all model results including path coefficients (maybe as appendix)

We added a figure including factor loading of all three defensible measurement model in the supporting information (S1 Fig).

Discussion:
- Because the authors repeatedly refer to differences in assessment (self-report vs. behavorial tasks) influencing correlational findings I recommend to discuss to what extent do the models and respective factors represent latent constructs in a narrower sense and method factors potentially qualifying the distinction of three dimensions and EF separately from Hot/Cool processes on a construct level.
--> although one central aim of the research was to identify a feasible measurement model in the ABCD context, the discussion should refer both to the implementation in research using ABCD data and to implications for research on EF/self-regulation in general together with respective assessment issues.
To some extent the finding of no general SR-factor represents the heterogeneity of approaches on self-regulation.

Thank you for this valuable suggestion. To emphasize the method-specificity of the EF and Cool factors, we revised the text to make this distinction clearer. Specifically, we added the following sentence in line 480–481:

‘The EF factor, therefore, does not necessarily represent a trait, but rather a method-specific representation of cool processes.‘

Additionally, we adapted the sentence in line 545–548 to clarify how method-specificity contributes to the lack of association between the factors:

‘This difference between context-specific, immediate control in the Hot factor and general, long-term control in the Cool factor, along with the more apparent issue of method-specificity, may explain the lack of association between Cool and EF, despite their close theoretical link.’

To address the broader implications of our findings beyond the ABCD study, we added a paragraph in lines 521–524:

‘Hence, our findings confirm the challenges of capturing constructs using different assessment modalities and raise questions whether SR is best represented as a general factor - particularly in small sample studies - also beyond the ABCD dataset.’

These revisions aim to clarify the influence of method-specificity on the factors and highlight the broader relevance of our findings for research on EF/self-regulation and assessment issues.

general remarks:
- the authors write about cold vs. hot processes/functions. For example, in Metcalfe & Mischel (1999) the common term is rather "cool" EF than "cold" EF. Following this, I recommend to change "cold" into "cool".

Thank you for this important remark. We exchanged the term ‘cold’ with ‘cool’ throughout the manuscript and the figures.

- sometimes, statements referring to previous research results have no reference, e.g. in

We added an additional reference in line 128 (‘Therefore, behavioral tasks may be more appropriate for studying brain-behavior relationships [29], which is one of the main objectives of the ABCD dataset.’), line 149 (‘Potentially related constructs were identified based on [2] and [30], […]’) and in line 495 (‘Academic achievement is considered to be associated with the Cool and EF factors more strongly as compared to the Hot factor (e.g., [32]).’).

- some English phrases seem a little bit uncommon, e.g.,: l. 72, l. 85, l.96-99
"exploit these data sets" in l. 43. Hence, I recommend to check English language again during revision.

We exchanged ‘exploit’ with ‘leverage’ in this sentence (line 52) and checked the language throughout the manuscript, especially in the paragraph ‘The measurement of self-regulation’ (lines 111-132) and parts of the discussion.

- I recommend to check what abbreviations are necessary and support text fluency and what abbreviations could be omitted to ensure that readers can easily understand the meaning of text content.

Please refer to our previous comment on abbreviations.

6. PLOS authors have the option to publish the peer review history of their article ([what does this mean?](https://track.editorialmanager.com/CL0/https:%2F%2Fjournals.plos.org%2Fplosone%2Fs%2Feditorial-and-peer-review-process%23loc-peer-review-history/1/010f0193b2fa538b-36a72850-d182-4b97-9558-d0d8e6c87070-000000/5Z_P21-SSYjfKZ58sLZNOpHRAa_TQXUXqEPS0kI9PQo=189)). If published, this will include your full peer review and any attached files.


If you choose “no”, your identity will remain anonymous but your review may still be made public.


**Do you want your identity to be public for this peer review?** For information about this choice, including consent withdrawal, please see our [Privacy Policy](https://track.editorialmanager.com/CL0/https:%2F%2Fwww.plos.org%2Fprivacy-policy/1/010f0193b2fa538b-36a72850-d182-4b97-9558-d0d8e6c87070-000000/S1KxXevBYoWk04S9u0SKAyMCXzxKyygn62wDe51E05o=189).

Reviewer #1: Yes: Julia Grass
