# Supplementary material for: The measurement of self-regulation in the Adolescent Brain Cognitive Development (ABCD) Study
Source: PLoS One. 2025 May 5;20(5):e0322795. doi: 10.1371/journal.pone.0322795 (PMC12052097; doi:10.1371/journal.pone.0322795)
Supplement: Attachment — Submitted filename: Response_to_Reviewers_auresp_2.docx [file pone.0322795.s005.docx]

Dear Prof. Dr. Iftikhar Ahmed Khan, dear Reviewer Dr. Grass,

Thank you for handling and reviewing our manuscript (PONE-D-24-12171R1) and for your insightful and positive feedback. We sincerely appreciate the constructive comments, which have once again helped us to improve the manuscript. We have carefully considered all suggestions and incorporated them into the revised version. Additionally, we have provided a version of the manuscript without the track changes.

Below, you will find our point-by-point response to the reviewers' comments, with line numbers referring to the final manuscript without track changes.

Yours sincerely,

Merle Marek, Axel Heep and Andrea Hildebrandt

**Journal Requirements:**

Please review your reference list to ensure that it is complete and correct. If you have cited papers that have been retracted, please include the rationale for doing so in the manuscript text, or remove these references and replace them with relevant current references. Any changes to the reference list should be mentioned in the rebuttal letter that accompanies your revised manuscript. If you need to cite a retracted article, indicate the article’s retracted status in the References list and also include a citation and full reference for the retraction notice.

We sincerely appreciate the editor’s careful observation. Upon reviewing the reference list, we confirmed that one cited publication had been retracted in the meantime:

*Schmidt, H., Daseking, M., Gawrilow, C., Karbach, J., & Kerner auch Koerner, J. (2022). RETRACTED: Self‐regulation in preschool: Are executive function and effortful control overlapping constructs? Developmental Science, 25(6), e13272.*

We have removed this reference from our article and replaced it with a more suitable source to support our assumptions:

*Allan, N. P., & Lonigan, C. J. (2014). Exploring dimensionality of effortful control using hot and cool tasks in a sample of preschool children. Journal of Experimental Child Psychology, 122, 33-47.*

Reviewers' comments:

Reviewer's Responses to Questions

**Comments to the Author**

1. If the authors have adequately addressed your comments raised in a previous round of review and you feel that this manuscript is now acceptable for publication, you may indicate that here to bypass the “Comments to the Author” section, enter your conflict of interest statement in the “Confidential to Editor” section, and submit your "Accept" recommendation.

Reviewer #1: All comments have been addressed

2. Is the manuscript technically sound, and do the data support the conclusions?

The manuscript must describe a technically sound piece of scientific research with data that supports the conclusions. Experiments must have been conducted rigorously, with appropriate controls, replication, and sample sizes. The conclusions must be drawn appropriately based on the data presented.

Reviewer #1: Yes

3. Has the statistical analysis been performed appropriately and rigorously?

Reviewer #1: Yes

4. Have the authors made all data underlying the findings in their manuscript fully available?

The [PLOS Data policy](http://track.editorialmanager.com/CL0/http:%2F%2Fwww.plosone.org%2Fstatic%2Fpolicies.action%23sharing/1/010f0195706af605-a5be197b-f330-4e83-8cf1-ff59660df014-000000/_aZv5PxE5z1DLoBhFsuiv0MGYSQjpw7fSWUr_8lrN-M=201) requires authors to make all data underlying the findings described in their manuscript fully available without restriction, with rare exception (please refer to the Data Availability Statement in the manuscript PDF file). The data should be provided as part of the manuscript or its supporting information, or deposited to a public repository. For example, in addition to summary statistics, the data points behind means, medians and variance measures should be available. If there are restrictions on publicly sharing data—e.g. participant privacy or use of data from a third party—those must be specified.

Reviewer #1: (No Response)

5. Is the manuscript presented in an intelligible fashion and written in standard English?

PLOS ONE does not copyedit accepted manuscripts, so the language in submitted articles must be clear, correct, and unambiguous. Any typographical or grammatical errors should be corrected at revision, so please note any specific errors here.

Reviewer #1: Yes

6. Review Comments to the Author

Please use the space provided to explain your answers to the questions above. You may also include additional comments for the author, including concerns about dual publication, research ethics, or publication ethics. (Please upload your review as an attachment if it exceeds 20,000 characters)

Reviewer #1: The authors have thoroughly revised and significantly improved the manuscript so that only minor remarks remain.

We thank the reviewer for the positive evaluation of our revision.

Open Data statement:
The new statement on data availability is transparent. Two minor remarks**:** Adding a link to the homepage, where data sharing restrictions by the ABCD-authors and the contact for requesting data for research purposes would be an improvement of transparency/clarity. The osf link is currently the anonymous one – when preparing the manuscript for publication, you should change it into the official one.

We have added a link to the webpage providing information on data access in line 339 and have also updated the OSF link.

Structure:
The revision of the introduction has improved the clarity and comprehensibility a lot. I understand the decision to introduce the ABCD study first bevor writing about self-regulation in general to prevent misleadingly focusing on SR in general. The authors have improved the clarity of the introduction and added important parts concerning study aims and theoretical information (e.g., definition) about core variables.

We appreciate your positive feedback on the revised introduction and are pleased that the restructuring and additional theoretical information have improved clarity and are now more in line with the aims of the study. Thank you for your thoughtful review and valuable suggestions, which have helped us to strengthen the manuscript.

Theory (Discussion)
The background for the tested models of SR became clearer including both theoretical and method-based reasoning for not including EF into cool functions. Maybe, this aspect would be valuable to include in the discussion briefly because distinguishing EF and cool functions refers to measures used in ABCD but is not theoretically generalizable to SR in general and other studies.

We have added such a discussion in lines 485-489:

‘Notably, distinguishing EF and Cool facets as separate factors is appropriate in the ABCD dataset due to the differences in measurement approaches. However, this distinction may not be theoretically generalizable to any psychometric study on SR, where method variance may be better controlled by the use of a more comprehensive multivariate assessment battery.’

Ll. 49-51: reference missing

We added three exemplary references regarding brain-behaviour associations in the domain of cognition, personality, and psychiatric disorders in line 51.

l. 58: I would extent the statement on the aim/benefit of the current research: providing a well-founded measurement model is not only advantageous for statistical analysis but also for drawing valid conclusions based on neuropsychological research on the ABCD study.

We extended the sentence in line 55-59 and rephrased it slightly to enhance clarity and readability.

‘Since researchers analyzing data from these neuroimaging initiatives often have expertise in neuroimaging but less so in psychometrics, proposing well-established and robust psychometric models—along with implementation code—can enhance the statistical analysis of brain-behavior associations and support valid conclusions in neuropsychological research using the ABCD study.’

ll. 113: The usage of “content domain” is slightly misleading although it corresponds with the term content validity. DoG-measures and EF measures refer to slightly different aspects of self-regulation but also to different measurement approaches.

We appreciate this observation and have revised the text to explicitly address both content and methodological domains. Specifically, we have reworded the sentence in lines 114-115 to clarify this distinction:

‘Research on the convergent validity of SR measures reveals weak correlations between different content- and methodological-domains [24,26].’

Additionally, to ensure that the overlap between content and methodological domains is acknowledged, we have added “from different content domains” in lines 131–133:

‘In light of these considerations, combining task-based and survey-based SR measures from different content domains poses both opportunities and risks.’

ll. 119: with the explanation of the reviewers I better understand why they have chosen to write about content validity instead of construct validity. However, because including measurement approaches (partly) referring to different construct facets and testing whether the measurement model represents one general latent construct (what somehow does include the idea of convergent validity), at some point they should refer also to construct validity and not only to content validity.

We believe that the whole measurement approach is guided by considerations of convergent validity. Therefore, we have restructured the paragraph ‘The measurement of self-regulation’ in lines 112-135 to more consistently and explicitly address problems with content- and also convergent validity, e.g., in lines 114-115:

‘Research on the convergent validity of SR measures reveals weak correlations between different content- and methodological domains [24,26].’

Or 121-123:

‘However, the consistently low correlations between survey and behavioral SR indicators [24] point to issues with convergent validity.’.

Methods:
Table 1: I recommend to add one brief statement in the table notes explaining that parenthetical information in the firs column refer sth. referring to the ABCD dataset (which is, what I suppose)

To enhance clarity, we added a statement

‘Parenthetical dataset names in the first column indicate the ABCD dataset-derived short names of the data structure from which the data was retrieved.’

in line 296-297 in addition to the column title ‘Test (ABD dataset name)’.

Minor remark: “lavaan” in line 308 seems to be written in another font than the rest of the text.

We adapted the font to the rest of the text.

Discussion:
ll. 545-548: hot and cool system do not mainly refer to long vs. short-term orientation but to the extent to which affect/emotions are involved or rather cognitively focused – mainly referring to long-/short term may be misleading because from a conceptual view basic EF are often considered to be part of the cool system without being long-term oriented per se (but of course helpful to reach long-term goals).

Thank you for pointing out this potential confusion. In that sentence, our intention was not to highlight differences between the Hot and Cool factors—given that they are correlated—but rather to explain the distinction between them in terms of contextualized versus decontextualized control. This distinction was meant to explain the lack of correlation between Cool and EF. The original wording was, however, misleading, so we have rephrased the sentence to achieve more clarity – see lines 554-559:

‘This dataset-specific representation of the Cool factor in terms of broad, decontextualized and long-term control mechanisms, along with the more obvious issue of method-specificity, may also explain the lack of association between Cool SR and EF, despite their close theoretical link. SR captured by behavioral task performance, as measured with EF, is less closely related to delayed (as captured by the Cool factor) than to immediate SR, as they operate on different temporal scales of SR. ‘

7. PLOS authors have the option to publish the peer review history of their article ([what does this mean?](https://track.editorialmanager.com/CL0/https:%2F%2Fjournals.plos.org%2Fplosone%2Fs%2Feditorial-and-peer-review-process%23loc-peer-review-history/1/010f0195706af605-a5be197b-f330-4e83-8cf1-ff59660df014-000000/eLuF9tc2bCmFdnNbkcQTLabyTE6Nfw9Wap-D1hyimIs=201)). If published, this will include your full peer review and any attached files.


If you choose “no”, your identity will remain anonymous but your review may still be made public.


**Do you want your identity to be public for this peer review?** For information about this choice, including consent withdrawal, please see our [Privacy Policy](https://track.editorialmanager.com/CL0/https:%2F%2Fwww.plos.org%2Fprivacy-policy/1/010f0195706af605-a5be197b-f330-4e83-8cf1-ff59660df014-000000/EoiJMVRsCJB0P2T3CP_L2RxE7l8DdAXGEUQyyHz_hf8=201).

Reviewer #1: **Yes:** Julia Grass

[NOTE: If reviewer comments were submitted as an attachment file, they will be attached to this email and accessible via the submission site. Please log into your account, locate the manuscript record, and check for the action link "View Attachments". If this link does not appear, there are no attachment files.]

While revising your submission, please upload your figure files to the Preflight Analysis and Conversion Engine (PACE) digital diagnostic tool, [https://pacev2.apexcovantage.com/](https://track.editorialmanager.com/CL0/https:%2F%2Fpacev2.apexcovantage.com%2F/1/010f0195706af605-a5be197b-f330-4e83-8cf1-ff59660df014-000000/2Sfk6HZdGkgy0eCnZ08xyAIEzcl2tyOf3ZJTzMGsrJw=201). PACE helps ensure that figures meet PLOS requirements. To use PACE, you must first register as a user. Registration is free. Then, login and navigate to the UPLOAD tab, where you will find detailed instructions on how to use the tool. If you encounter any issues or have any questions when using PACE, please email PLOS at [figures@plos.org](mailto:figures@plos.org). Please note that Supporting Information files do not need this step.

We have uploaded the files and PACE indicated that they comply with the PLOS requirements.
